# Evaluating the therapeutic effect of different forms of silymarin on liver status and expression of some genes involved in fat metabolism, antioxidants and anti‐inflammatory in older laying hens

**DOI:** 10.1002/vms3.70025

**Published:** 2024-09-26

**Authors:** Samira Faryadi, Ardashir Sheikhahmadi, Ayoub Farhadi, Himan Nourbakhsh

**Affiliations:** ^1^ Department of Animal Science Faculty of Agricultural University of Kurdistan Sanandaj Iran; ^2^ Department of Animal Science Faculty of Animal Sciences and Fisheries Sari Agricultural Sciences and Natural Resources University Sari Iran; ^3^ Department of Food Science and Engineering Faculty of Agriculture University of Kurdistan Sanandaj Iran

**Keywords:** hepatic steatosis, lecithin, laying hens, nano‐silymarin

## Abstract

**Background:**

Silymarin, the predominant compound of milk thistle, is an extract took out from milk thistle (*Silybum marianum*) seeds, containing a mixture of flavonolignans with strong antioxidant capability.

**Methods:**

The experiment was conducted using 70 Lohmann LSL‐Lite hens at 80 weeks of age with 7 treatments each with 10 replicates. Treatments included: (1) control diet without silymarin, (2) daily intake of 100 mg silymarin powder/kg body weight (BW) (PSM100), (3) daily intake of 200 mg silymarin powder/kg BW (PSM200), (4) daily intake of 100 mg nano‐silymarin/kg BW (NSM100), (5) daily intake of 200 mg nano‐silymarin/kg BW (NSM200), (6) daily intake of 100 mg lecithinized silymarin/kg BW (LSM100) and (7) daily intake of 200 mg lecithinized silymarin/kg BW (LSM200). The birds were housed individually, and diets were fed for 12 weeks.

**Results:**

Scanning electron microscopy showed that NSM was produced with the average particle size of 20.30 nm. Silymarin treatment improved serum antioxidant enzyme activity. All groups receiving silymarin showed a decrease in liver malondialdehyde content, expression of fatty acid synthase, tumour necrosis factor alpha, interleukin 6 (IL‐6) genes in the liver, and hepatic steatosis than the control, except those fed the PSM100 diet. There were decreases in liver dry matter and fat contents, non‐alcoholic fatty liver disease and hepatocyte ballooning, and an increase in glutathione peroxidase gene expression and a decrease in iNOS gene expression in birds fed the NSM100, NSM200, LSM100 and LSM200 diets compared to the control group. Moreover, all groups receiving silymarin showed a significant decrease in liver weight compare to the control group.

**Conclusions:**

Overall, the effects of silymarin when converted to NSM or LSM and offered at the level of 200 mg/kg BW were more pronounced on the hepatic variables and may be useful in the prevention of the liver disease in older laying hens.

## Graphical abstract

Silymarin improved serum antioxidant enzyme activity.

Silymarin showed a decrease in liver malondialdehyde content, expression of FASN, TNFα, IL‐6, iNOS genes in liver and hepatic steatosis.

Silymarin decreased liver dry matter and fat content, non‐alcoholic fatty liver disease and hepatocyte ballooning, liver weight and an increase in GPx gene expression.

Silymarin when converted to NSM or LSM and offered at the highest level of 200 mg/kg BW were more pronounced on the hepatic variables.

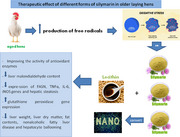

## 1 INTRODUCTION

The laying hens experience a gradual decline in their production performance after a temporary period of high egg production rate and their peak laying stage (Tumova et al., 2017). This happens for various reasons from age‐related changes in the digestive and reproductive system (Gu et al., 2021; Peebles et al., 2006), internal metabolic pathways (Wang et al., 2019), intestinal microbial flora (Wang et al., 2020) and so on. Among them, lipid metabolism plays an important role in the process of egg formation and is also closely related to the molecular factors and physiological characteristics of the liver, the primary metabolic organ for laying hens (Gloux et al., 2019). It has been reported that cell volume reduction and cytoplasmic lipofuscin accumulation are common structural changes that occur naturally in ageing hepatocytes. Lipofuscin is a highly oxidized and cross‐linked protein that can induce oxidative stress (Pinto et al., 2020). Moreover, lipid metabolism disorders and related metabolic diseases can be exacerbated by hepatic stress and liver damage during the ageing process (Xiong et al., 2014). Furthermore, it has been reported that an age‐specific pattern of genes and proteins involved in hepatic lipid metabolism could explain the underlying mechanism of fatty liver, which has a high prevalence in the individuals (Gu et al., 2021). Ageing is a natural physiological process that can gradually generate harmful reactive oxygen species (ROS) (Lee et al., 2004). As a result, when endogenous antioxidants are not enough to neutralize excessive free radicals and peroxides in organisms, redox homeostasis is disrupted and oxidative stress occurs (Estevez, 2015). Thus, during ageing, ROS inducers (e.g. hydrogen peroxide) can increase the synthesis and accumulation of lipids along with the up‐regulated mRNA levels of genes related to cholesterol synthesis and absorption‐in hepatocytes, indicating the negative effects of age‐related oxidative stress on fat deposition (Seo et al., 2019). For domestic laying hens, it has been found that reduced egg production rate is related to reduced hepatic yolk precursors (Liu et al., 2018). Therefore, as older laying hens are more vulnerable to internal and external stimulation than younger ones, there is considerable published literature focusing on the ameliorating effects of bioactive additives on the reproductive performance and physiological performance of older laying hens (Jia et al., 2016; Liu et al., 2020).

Therefore, the need for antioxidants that can inhibit the formation of free radicals increases in order to control and reduce lipid peroxidation (Kehrer, 1993). Botsoglou et al. (2005) reported the effectiveness of herbs and spices antioxidant components to prevent lipid oxidation in animal products. Silymarin, the predominant compound of milk thistle, is an extract brought out from milk thistle (*Silybum marianum*) seeds, containing a mixture of flavonolignans with strong antioxidant capability (Saeed et al., 2017). Some of the hepato‐protective activities of silymarin reported by Salomone et al. (2016) include modulating the immune system, inhibiting free radicals and restoring the function of antioxidative enzymes such as glutathione concentration, reducing oxidative stress, anti‐fibrotic, anti‐inflammatory effects and generating cell membrane stabilization. So, silymarin has a high potential to be a valuable antioxidant to prevent the production of free radicals and lipid peroxidation; however, the low intestinal absorption limits its availability for entry into the body. There are several methods to increase the solubility and bioavailability of a low water‐soluble drug. The bioavailability of silybin increases 10‐fold when complexed with phosphatidylcholine (Morazzoni et al., 1993). In this context, Tedesco et al. (2004) showed that the silymarin‐phospholipid complex protects broilers against the harmful effects of aflatoxin B_1_. Soy lecithin is a mixture of various phospholipids such as phosphatidylcholine, phosphatidylethanolamine and phosphatidylinositol (Akit et al., 2016), which has been reported as a suitable compound to increase intestinal absorption of silymarin (Wang et al., 2015). Moreover, during the past decades, nanotechnology methods have been used to improve the bioavailability of silymarin in new pharmaceutical applications (Di Costanzo & Angelico, 2019). Kakran et al. (2015) reported that the nanosuspension evaporative deposition method is an effective method for making pharmaceutical nanoparticles with an increased dissolution rate. Parveen et al. (2011) reported that the use of silymarin nanoemulsion in low doses has similar or higher effects than a higher dose of silymarin, which indicates improved bioavailability and better absorption of poorly water‐soluble substances in the form of nanoemulsion. In this context, Hajizadeh Moghaddam et al. (2017) reported that silymarin and its nano‐crystals probably protect the liver against damage caused by the consumption of titanium oxide nanoparticles due to their antioxidant properties. Nutritional and antioxidant effects of nano‐silymarin and silymarin‐phosphatidylcholine complex have been investigated in rodents (Di Costanzo & Angelico, 2019; Fatehi et al., 2018; Ustyol et al., 2017). However, no data about their potential use in laying hens or other poultry have been published in the literature. So, in the present study, we produced nanoparticles of silymarin by the evaporative deposition method of nano‐suspension (Kakran et al., 2015) and then experimentally compared whether the dietary supplementation of silymarin, nano‐silymarin and silymarin‐lecithin complex to laying hens had a protective effect on liver status and expression of some genes involved in fat metabolism, antioxidants and anti‐inflammatory in older laying hens.

## 2 MATERIALS AND METHODS

### 2.1 Preparation of silymarin and nano‐silymarin

**FIGURE 1:**
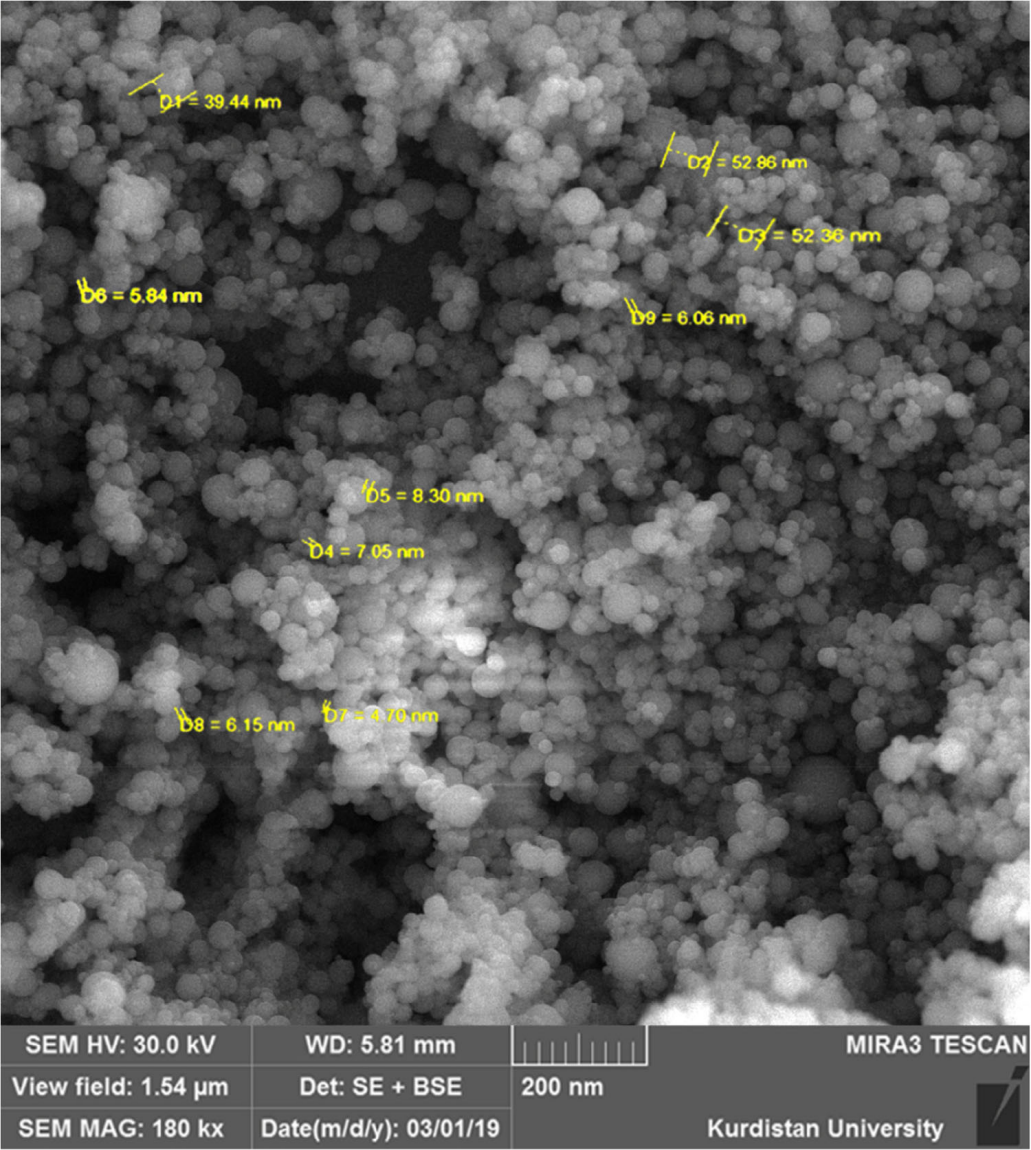
Scanning electron microscopy of nano‐silymarin: The mean particle size is 20.30 nm.

Silymarin powder with a purity of 80.18% was purchased from Zard Band Pharmaceutical Company. Nano‐silymarin crystals were prepared using the evaporative deposition method of nano‐suspension as described by Kakran et al. (2015) with some modifications. Briefly, 2 mg of silymarin was dissolved in 1 mL of acetone (solvent) and put in ultrasonication for 1 h. Then hexane (anti‐solvent) was added to prepare a nano‐suspension. The ratio of solvent to anti‐solvent was 1:30. The obtained nano‐suspension was placed in a water bath at a 40°C and set to a rotation of 90 rpm for 45 min. After evaporation, the solid particles were collected in a flask and used for further analysis. The size of the particles was measured using a scanning electron microscope (TESCAN MIRA3, sro Brno, Figure 1).

### 2.2 Birds, management and treatments

Seventy laying hens (Lohmann LSL‐Lite) at 80 weeks of age with similar body weight (BW) (1650 ± 74 g) were individually allocated to 7 treatments with 10 replicates cages of 1 hen per treatment for 12 weeks. Birds did not have any access to the feed of each other because each cage had an individual feeder and a nipple waterer. The birds were fed with experimental treatments included: (1) control diet without silymarin, (2) daily intake of 100 mg silymarin powder/kg BW (PSM100), (3) daily intake of 200 mg silymarin powder/kg BW (PSM200), (4) daily intake of 100 mg nano‐silymarin/kg BW (NSM100), (5) daily intake of 200 mg nano‐silymarin/kg BW (NSM200), (6) daily intake of 100 mg lecithinized silymarin/kg BW (LSM100) and (7) daily intake of 200 mg lecithinized silymarin/kg BW (LSM200). The ratio of 1:2 silymarin and lecithin was used to prepare the silymarin‐lecithin complex (Tedesco et al., 2004). Feed (Table 1) and water were supplied ad libitum. Birds were fed daily 115 g of control diet, which was considered for each bird (each cage). First, the amount of silymarin per kg of BW/day was calculated for each bird. Then, the amount obtained was calculated for 7 days of the week and added to the feed intended for the same week (based on 115 g/day) and completely mixed by hand. This work was done for all the cages individually and kept in special buckets for each cage. During the day, the intended amount for each bird (115 g) was given to the birds twice. It should be noted that feed intake was ad libitum, and this was done to minimize feed loss and ensure that the bird received its intended daily dose of silymarin. The temperature in the house was 20°C, and the lighting program was set at 16 h of light and 8 h of dark.

**TABLE 1:** Composition of the basal diet.

Item	Quantity (g/kg, as noted)
Ingredients	
Corn	658.3
Soybean meal (44% crude protein)	223.9
Soybean oil	1.70
Limestone	93.9
Dicalcium phosphate	11.6
Common salt	1.80
Vitamin–mineral premix^a^	5
dl‐Methionine	1.2
Sodium bicarbonate	2.2
Calculated composition	
Metabolizable energy (kcal/kg)	2725
Crude protein	146.4
Calcium	39.1
Available phosphorous	3.30
Sodium	1.5
Methionine	3.5
Methionine + cysteine	6.9
Lysine	7.5

^a^ The vitamin–mineral premix provided the following quantities per kg of diet: retinyl acetate, 2.70 mg; cholecalciferol, 0.05 mg; α‐tocopherol acetate, 18 mg; menadione, 2 mg; thiamine, 1.8 mg; riboflavin, 6.6 mg; niacin, 10 mg; pantothenic acid, 30 mg; pyridoxine, 3 mg; folic acid, 1 mg; cobalamin, 0.015 mg; biotin, 0.1 mg; choline chloride, 250 mg; manganese (as MnSO_4_·H_2_O), 105 mg; iron (as FeSO_4_·7H_2_O), 38 mg; zinc (as ZnO), 126 mg; copper (as CuSO_4_), 16 mg; selenium (as Na_2_SeO_3_), 0.28 mg; iodine (as Ca(IO_3_)_2_), 1.0 mg.

### 2.3 Liver histopathology

2.3

At the end of the experiment, four birds from each treatment were randomly selected and killed by severing the jugular vein. The liver was removed and weighed, and then the organ weight was expressed as a percentage of live BW. At the time of slaughter, liver tissue samples were taken from the right lobe of each hen, and for histopathological evaluation, they were first placed in 5% formalin solution for 24 h, and then the samples were fixed in 10% neutral buffered formalin, embedded in paraffin, sectioned at 5 µm thicknesses and stained with haematoxylin–eosin for light microscopic examination. Description and scoring of steatosis were performed according to the grading criteria consistent with the NASH Clinical Research Network Scoring System (Davis et al., 2016). Briefly, steatohepatitis was graded based on the extent of parenchyma involved (Grade 0, <5% of hepatocytes; Grade 1, <33% of hepatocytes; Grade 2, 33%–66% of hepatocytes; Grade 3, >66% of hepatocytes). Hepatocellular ballooning was evaluated for zonal location, and an estimate of severity (Grade 0, none; Grade 1, mild and Grade 2, marked) was made based on numbers of hepatocytes showing this change. Finally, a composite NAFLD activity score (NAS) was calculated as the sum of these individual measures.

### 2.4 liver dry matter and fat content

The percentage of liver dry matter was determined by placing the samples (four samples per treatment) in an oven at 105°C for 24 h. Liver samples were ground using a mortar. Then, 1 g of the ground liver was poured into filter paper and placed in a Soxhlet‐type extractor for 24 h using petroleum ether with a boiling point of 60–70°C to extract its fat. Then the fat weight of each sample was calculated based on dry weight.

### 2.5 Serum activity of hepatic enzymes and liver malondialdehyde concentration

At the end of 92 weeks of age, four birds from each treatment were randomly selected and individually weighed, and then blood samples were taken from the wing vein using syringes to separate serum and transferred into tubes that were EDTA‐free. Blood samples were centrifuged (3000 rpm, 15 min, 4°C), and the serum was separated and stored at −20°C for further analysis of the activities of aspartate aminotransferase (AST), alanine aminotransferase (ALT), alkaline phosphatase (ALP), gamma‐glutamyl transferase (GGT), using commercial kits (Pars Azmun).

To assess malondialdehyde (MDA) contents, the liver samples (5 g of fresh liver) were homogenized in a buffer solution (1.15% potassium chloride, pH 7.4) at refrigerated temperature. Homogenized samples were centrifuged for 15 min at 5000 × *g*, and the supernatant was taken and used to measure MDA. Total antioxidant capacity (TAC) was determined using the Randox total antioxidant status kit (Randox Laboratories Ltd.). The basis of the measurement of serum MDA is based on the reaction with thiobarbituric acid, extraction with normal butanol, absorbance measurements by spectrophotometric method at 532 nm and comparison of absorption with the standard curve.

### 2.6 Measurement of relative expression of acetyl‐CoA carboxylase (ACACA), fatty acid synthase (FASN), glutathione peroxidase (GPx), interleukin 6 (IL‐6), iNOS and tumour necrosis factor alpha (TNF‐α) genes in the liver

To measure the relative expression of genes, liver samples were collected from the right lobe (four samples per treatment) and immediately placed in liquid nitrogen. RNA samples were extracted using RNeasy mini kit (QIAGEN, 74104) and converted to cDNA using a cDNA synthesis kit (QIAGEN, 205311). The relative expression of genes was determined by real‐time PCR using specific primer pairs (Table 2) and Quantifast SYBR Green PCR kit (QIAGEN, 204052) in Corbett PCR device (Corbett, Rotor gene 3000). GAPDH gene was used as an internal control gene.

**TABLE 2:** Primer sequences of tumour necrosis factor alpha (TNF‐α), interleukin 6 (IL‐6), acetyl‐CoA carboxylase (ACACA), fatty acid synthase (FASN), glutathione peroxidase (GPx), iNOS and GAPDH (GG)

Gene	Primers sequences (5′–3′)	Temp. (°C)	Size (bp)	Accession number
TNF‐α	F‐GACAGCCTATGCCAACAAGTA R‐GAATTAAGCAACAGCCAGCTATG	60	213	AY765397.1
IL‐6	F‐AGCGAAAAGCAGAACGTCGAGTC R‐GCCGAGTCTGGGATGACCACTTC	60	107	XM_015281283.2
ACACA	F‐CAGATTTGTTGTCATGGTGAC R‐ACAGCCTGCACTGGAATGC	60	162	NC_006106
FASN	F‐CCACGTGTCAGTGTCAGAGA R‐GGCAGCAGAGCAGATGAGAT	60	136	NM_205155
GPx	F‐TCGAGAAGTGCGAGGTGAAC R‐GTACTGCGGGTTGGTCATCA	60	113	NM_001277853.3
iNOS	F‐ATTGTGGAAGGACCGAGCTG R‐CCTCGCACACGGTACTCATT	60	141	NM_204961.2
GAPDH (GG)	F‐CTGCCGTCCTCTCTGGC R‐GACAGTGCCCTTGAAGTG	60	119	NM_204305

The qPCR reaction mixture was prepared in 15 µL containing 7 µL SYBR Green, 1 µL of each forward and reverse primers, 1 µL cDNA and 5 µL deionized water. The qPCR thermal cycling included 1 cycle for 5 min at 95°C followed by 40 cycles of 30 s at 95°C, 30 s at 60°C and 45 s at 72°C.

### 2.7 Statistical analysis

Data were analysed as a 3 × 2 factorial + 1 using the general linear model procedure of SAS software (SAS Institute Inc.). The statistical model included the form of silymarin (PSM, NSM and LSM), added silymarin level (100 and 200 mg/kg BW), and their interaction. When differences were significant, means were separated using Tukey's studentized range tests. The histopathology data were analysed with non‐parametric method by Npar 1 way procedure of SAS software. All statements of significance were based on *p* < 0.05.

## 3 RESULTS

### 3.1 Serum antioxidant enzyme activity

The effects of different forms and levels of silymarin on serum antioxidant enzyme activity are shown in Table 3. The activity of ALP was lower in birds fed the NSM100 and NSM200 than other dietary treatments (*p* < 0.05). All groups receiving silymarin showed a decrease in the serum MDA concentration and an increase in TAC than the control group (*p* < 0.05) except those fed the PSM100 diet. Moreover, the activity of ALT in all experimental groups and the activity of AST in the group containing NSM200 were lower than the control group (*p* < 0.05). However, the serum GGT activity decreased in the hens fed the NSM100, NSM200 and LSM200 diets compared to the control group (*p* < 0.05). Moreover, the effects of silymarin on MDA concentration, GGT activity and TAC were more pronounced when converted to NSM or LSM (*p* < 0.05), and similar values were observed with the consumption of silymarin at both levels of 100 and 200 mg/kg BW (*p* < 0.05).

**TABLE 3:** The effect of different forms and levels of silymarin on serum antioxidant enzyme activity of older laying hens.

	Dietary treatments (DT)																
		PSM		NSM		LSM		Type (T)			Level (L)			*p*‐Value			
Item	Control	100	200	100	200	100	200	PSM	NSM	LSM	100	200	SEM	DT	T	L	T × L
ALP	497.2^a^	412.7^ab^	346.3^ab^	316.3^b^	312.7^b^	330.6^ab^	327.2^ab^	379.5	314.5	328.9	353.2	328.7	18.09	0.02	0.1	0.3	0.5
AST	225.0^a^	214.2^ab^	206.2^ab^	204.2^ab^	197.4^b^	201.4^ab^	200.2^ab^	210.2	200.8	200.8	206.6	201.2	2.49	0.02	0.1	0.2	0.7
GGT	42.7^a^	40.4^ab^	39.4^ab^	38.2^b^	36.7^b^	38.4^b^	37.8^b^	39.9^a^	37.4^b^	38.1^ab^	39.0	38.0	0.45	0.001	0.04	0.1	0.8
ALA	15.7^a^	14.1^b^	13.2^b^	13.2^b^	12.9^b^	13.1^b^	13.1^b^	13.6	13.0	13.1	13.5	13.0	0.24	0.008	0.4	0.3	0.6
MDA	2.8^a^	2.5^ab^	2.1^bc^	1.8^c^	1.7^c^	1.9^bc^	1.9^bc^	2.3^a^	1.7^b^	1.9^ab^	2.1	1.9	0.09	0.007	0.01	0.2	0.5
TAC	0.94^c^	1.0^bc^	1.6^ab^	1.8^a^	1.9^a^	1.6^ab^	1.7^a^	1.3^b^	1.9^a^	1.7^ab^	1.5	1.7	0.09	0.009	0.04	0.1	0.5

*Note*: Within DT, T and L and in each row means with different superscripts letters (a–c) are significantly different (*p* < 0.05).

Abbreviations: ALA, alanine aminotransferase (U/L); ALP, alkaline phosphatase (U/L); AST, aspartate aminotransferase (U/L); GGT, gamma‐glutamyl transferase (U/L); LSM, lecithinized silymarin; MDA, malondialdehyde (nmol/L); NSM, nano‐silymarin; PSM, powdered silymarin; SEM, standard error of means; TAC, total antioxidant capacity (nmol/L).

### 3.2 Hepatic variables

The effect of different forms and levels of silymarin on liver weight, dry matter and fat contents, and hepatic MDA levels are shown in Table 4. There were decreases in liver dry matter and fat contents in hens fed the NSM100, NSM200, LSM100 and LSM200 diets compared to the control group (*p* < 0.05). All groups receiving silymarin showed a significant decrease in liver weight than the control (*p* < 0.05). It should be noted that type of silymarin did not affect any of these parameters. In addition, a decrease in liver MDA content (*p* < 0.05) was observed in all groups receiving silymarin except those fed the PSM100 diet. Moreover, the liver MDA content decreased upon an increase in the silymarin level and by passing from the PSM to the NSM and LSM diets (*p* < 0.05).

**TABLE 4:** The effect of different forms and levels of silymarin on hepatic variables of older laying hens.

	Dietary treatments (DT)																
		PSM		NSM		LSM		Type (T)			Level (L)			*p*‐Value			
Item	Control	100	200	100	200	100	200	PSM	NSM	LSM	100	200	SEM	DT	T	L	T × L
Dry matter (%)	41.7^a^	39.6^ab^	36.1^ab^	35.1^b^	34.1^b^	35.3^b^	34.1^b^	38.1	34.6	34.6	36.8	34.6	0.8	0.04	0.2	0.2	0.8
Fat content (%)	27.7^a^	25.1^ab^	23.5^ab^	21.7^b^	21.4^b^	22.7^b^	21.5^b^	24.3	21.6	22.1	23.0	22.0	0.6	0.04	0.1	0.3	0.8
Liver weight (%)	2.9^a^	2.4^b^	2.4^b^	2.3^b^	2.3^b^	2.3^b^	2.3^b^	2.4	2.3	2.3	2.3	2.3	0.06	0.04	0.8	0.8	0.9
MDA	2.5^a^	2.3^ab^	1.9^bc^	1.8^c^	1.5^c^	1.8^c^	1.5^c^	2.1^a^	1.6^b^	1.6^b^	2.0^a^	1.6^b^	0.08	0.0002	0.001	0.006	0.8

*Note*: Within DT, T and L and in each row means with different superscripts letters (a–c) are significantly different (*p* < 0.05).

Abbreviations: LSM, lecithinized silymarin; MDA, malondialdehyde (nmol/mg protein); NSM, nano‐silymarin; PSM, powdered silymarin; SEM, standard error of means.

### 3.3 Expression of liver genes of ACACA, FASN, GPx, IL‐6, TNF‐α and iNOS

The relative hepatic mRNA expressions of acetyl‐CoA carboxylase (ACACA), fatty acid synthase (FASN), glutathione peroxidase (GPx), interleukin 6 (IL‐6) and tumour necrosis factor alpha (TNF‐α) are reported in Table 5. There was a decrease in the expression of liver FASN, in groups receiving PSM200, NSM100, NSM200, LSM100 and LSM200 diet than the control (*p* < 0.05) and those fed the PSM100 diet. Moreover, IL‐6 gene expression decreased in birds fed diets containing PSM200, NSM100, NSM200, LSM100 and LSM200 diets compared to the control group and PSM100 (*p* < 0.05), although PSM200 was numerically reduced compared to PSM100. All groups receiving silymarin showed a decrease in TNF‐α gene expression (*p* < 0.05) except those fed the PSM100 diet. But the treatment containing NSM200 and LSM200 showed greater reduction than other treatments. However, there was an increase in GPx gene expression and a decrease in iNOS gene expression in birds fed diets containing NSM100, NSM200, LSM100 and LSM200 diets compared to the control group (*p* < 0.05). In addition, FASN, IL‐6, TNF‐α and iNOS showed lower concentrations, whereas GPx showed higher concentration with 200 mg/kg of silymarin as LSM or NSM than PSM (*p* < 0.05).

**TABLE 5:** The effect of different forms and levels of silymarin on expression of genes involved in fat metabolism, antioxidants and anti‐inflammatory of older laying hens.

	Dietary treatments (DT)																
		PSM		NSM		LSM		Type (T)			Level (L)			*p*‐Value			
Item	Control	100	200	100	200	100	200	PSM	NSM	LSM	100	200	SEM	DT	T	L	T × L
ACACA	2.5	1.8	1.5	1.0	0.9	1.0	0.9	1.7	0.9	0.9	1.2	1.1	0.2	0.2	0.1	0.6	0.9
FASN	2.3^a^	2.1^a^	1.1^b^	0.9^b^	0.8^b^	0.9^b^	0.8^b^	1.6^a^	0.8^b^	0.9^b^	1.3^a^	0.9^b^	0.1	0.0001	0.001	0.01	0.06
GPx	1.4^d^	1.7^d^	2.0^cd^	2.8^abc^	3.6^a^	2.6^bc^	3.2^ab^	1.8^b^	3.2^a^	2.9^a^	2.3^b^	2.9^a^	0.1	0.0001	0.0006	0.03	0.6
IL‐6	1.9^a^	1.7^ab^	1.3^bc^	1.1^c^	0.9^c^	1.0^c^	1.0^c^	1.5^a^	1.0^b^	1.0^b^	1.3	1.1	0.08	0.001	0.02	0.1	0.5
TNF‐α	1.0^a^	0.8^ab^	0.6^bc^	0.5^bcd^	0.3^d^	0.6^bc^	0.4^cd^	0.7^a^	0.4^b^	0.5^ab^	0.6^a^	0.4^b^	0.05	0.001	0.02	0.02	0.7
iNOS	2.0^a^	1.8^ab^	1.5^abc^	1.2^bcd^	0.8^d^	1.1^cd^	0.9^d^	1.7^a^	1.0^b^	1.0^b^	1.4^a^	1.1^b^	0.1	0.0009	0.002	0.04	0.8

*Note*: Within DT, T and L and in each row means with different superscripts letters (a–c) are significantly different (*p* < 0.05).

Abbreviations: ACACA, acetyl‐CoA carboxylase; FASN, fatty acid synthase; GPx, glutathione peroxidase; IL‐6, interleukin 6; LSM, lecithinized silymarin; NSM, nano‐silymarin; PSM, powdered silymarin; SEM, standard error of means; TNF‐α, tumour necrosis factor alpha.

### 3.4 Liver histopathological observations

**FIGURE 2:**
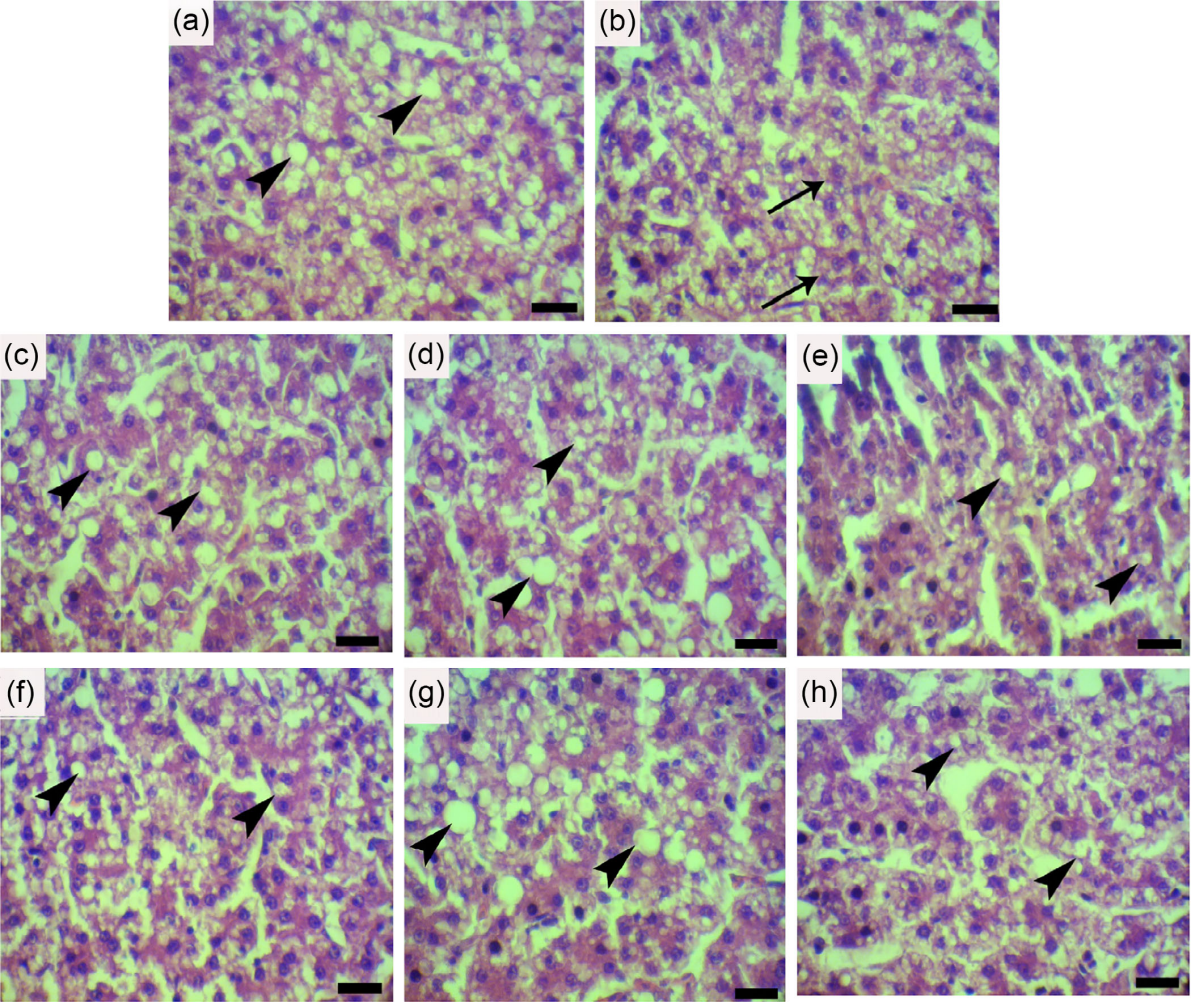
Haematoxylin and eosin staining of hepatic tissue from the hens in different groups (magnification bar = 25 µm). Severe macrovesicular steatosis (arrowheads) in control group (a); Ballooning degeneration (arrows) in control group (b); macrovesicular steatosis (arrowheads) in PSM_100_ group (c); PSM_200_ group (d); LSM_100_ group (e); LSM_200_ group (f); NSM_100_ group (g); NSM_200_ group (h).

Histopathological changes in the liver of hens studied in this experiment are shown in Figure 2 and Table 6. The degree of macrovesicular steatosis was highly variable, from mild in NSM and LSM treatments at both levels of administration and PSM at the level of 200 mg/kg BW to severe in the control group (*p* < 0.05). The vacuolar change was often patchy and more heavily focused in the centrilobular areas. The vacuole size and number ranged from numerous small pinpoint vacuoles to large single vacuoles prominently distending the hepatocyte cytoplasm. Collectively, the lowest score of hepatic steatosis and hepatocyte ballooning was observed in the NSM200 group, followed by NSM100, LSM200, LSM100, PSM200 and PSM200 groups, respectively (*p* < 0.05). The score of NAFLD activity was significantly lower in NSM100, NSM200, LSM100 and NSM200 groups than that in the control group (*p* < 0.05). There was no evidence of inflammation and fibrosis in all dietary groups. Generally, in comparison between treated groups, the most therapeutic effects were observed with the administration of NSM treatment at the level of 200 mg/kg BW (*p* < 0.05).

**TABLE 6:** The effect of different forms and levels of silymarin on liver histopathology of older laying hens.

	Dietary treatments (DT)																
		PSM		NSM		LSM		Type (T)			Level (L)			*p*‐Value			
Item	Control	100	200	100	200	100	200	PSM	NSM	LSM	100	200	SEM	DT	T	L	T × L
NAFLD	3.6^a^	3.0^ab^	2.8^abc^	2.0^cde^	1.3^e^	2.6^bcd^	1.9^ed^	2.9^a^	1.7^c^	2.3^b^	2.6^a^	2.0^b^	0.1	0.0001	0.0001	0.01	0.6
Hepatic Steatosis	2.3^a^	2.0^ab^	1.8^b^	1.4^c^	0.9^d^	1.8^b^	1.3^c^	1.9^a^	1.1^c^	1.5^b^	1.7^a^	1.3^b^	0.06	0.0001	0.0001	0.002	0.3
Hepatocyte ballooning	1.2^a^	1.0^ab^	0.9^abc^	0.6^bc^	0.4^c^	0.8^abc^	0.6^bc^	1.0^a^	0.5^b^	0.7^ab^	0.8	0.6	0.06	0.01	0.01	0.2	0.9

*Note*: Within DT, T and L and in each row means with different superscripts letters (a–c) are significantly different (*p* < 0.05).

Abbreviations: LSM, lecithinized silymarin; NAFLD, non‐alcoholic fatty liver disease; NSM, nano‐silymarin; PSM, powdered silymarin; SEM, standard error of means.

## 4 DISCUSSION

According to the results of scanning electron microscopy (Figure 1), it was found that silymarin nanoparticles were produced, and their average particle size was 20.30 nm. These particles were obtained with a concentration of 2 mg/mL and a solvent to anti‐solvent ratio of 1:30. The particle size becomes smaller by decreasing the concentration of substances with low solubility in water and increasing the ratio of solvent to anti‐solvent. The reason for this can be explained by the formation of nano‐particles via a homogeneous nucleus (Faryadi et al., 2021). These conditions may lead to higher super saturation and the formation of a large number of nuclei (Sharif & Elgayar, 2013). Increasing the number of nuclei means increasing in the number of nuclei with a smaller size. Therefore, in the present study, after trial and error with several different concentrations, we were able to use low concentrations of silymarin (2 mg/mL) and a high ratio of solvent to anti‐solvent (1:30) to make silymarin nanoparticles.

In vitro and in vivo studies in cell and mouse models show that age is closely related to metabolism and organ function (Allaire & Gilgenkrantz, 2020; Gong et al., 2017). Gu et al. (2021) reported that aged hens had higher AST activity but lower protein levels (total protein and globulin), suggesting that the aged livers may be damaged (Hanley et al., 2004) and have poor protein synthesis efficiency (Papet et al., 2003). Liver is the primary metabolic organ for laying hens (Li, Wang, et al., 2015), and these changes may affect material synthesis and transport through multiple pathways. In addition, Gu et al. (2021) reported that aged birds have higher concentrations of triglycerides and cholesterol in their livers. In this regard, there is a report that ageing can increase the accumulation of lipid in the liver and white adipose tissues by increasing plasma insulin concentration in rodent models (Honma et al., 2011). Excessive fat accumulation in the liver is responsible for metabolic syndrome and diseases and thus can affect the growth and reproductive performance of animals (Moradi et al., 2013; Shini et al. 2019). Additionally, Gu et al. (2021) found that aged bird shows lower circulating concentrations of oestradiol, which is a major class of oestrogens and is secreted from the ovarian follicles of laying hens. This change may indicate the degenerative state of reproductive organs in old birds. Oestradiol can affect lipid precursor formation by binding to specific receptors in the liver (Liu et al., 2018). It has been reported that oestradiol can up‐regulate the expression of long non‐coding RNAs associated with hepatic lipid metabolism, particularly for triglyceride biosynthesis and transport (Li et al., 2018). In addition, circulating oestradiol can act as a regulator of gene expression related to antioxidant and redox biology, and its reduction may lead to a weakened antioxidant system in older individuals (Bllanti et al., 2013). Oxidative stress has been considered a key factor in lipid accumulation and liver damage through complex signal transduction pathways (Li, Tan, et al., 2015; Zhao et al., 2020). It has been shown that ageing can stimulate lipid peroxidation and protein oxidation, leading to delays in the production and recovery of antioxidants, suppression of hepatocyte proliferation and liver tissue repair (Cheng et al., 2017; Tanimizu et al., 2020). Commercial laying hens produce large amounts of free radicals due to maintenance in the cage and high egg production. However, the ability to remove free radicals gradually decreases over time as increased bird's age. Oxidative stress and inflammatory responses are recognized as important mechanisms in the development and progression of non‐alcoholic fatty liver disease (Malaguarnera et al., 2009). Therefore, the therapeutic effect of different forms of silymarin on antioxidant capacity, liver status and expression of some genes involved in fat metabolism was measured to evaluate the liver function of old laying hens in the present study. The results showed that silymarin supplements improve serum antioxidant enzyme activity. High concentrations of transaminases are present in hepatocytes and hepatic parenchyma cells. The leakage of these enzymes into the bloodstream indicates the destruction of liver cells. Therefore, increasing the level of liver enzymes in the serum is one of the main indicators of liver damage (Hertog & Hollmann, 1996). The effect of silymarin on hepatic transaminases has been reported. Silymarin can treat damaged liver cells and restore normal liver function (Abenavoli et al., 2010). It is reported that administration of silymarin and silybin to rats has resulted in inhibition of the activity of liver enzymes such as GGT, ALT and AST (Wang et al., 1996). Baradaran et al. (2019) reported that the use of silymarin (100 mg/kg BW) in the diet reduces MDA content and TAC in broiler chicken exposed to carbon tetrachloride. Yi et al. (2012) showed that although oxidative stress increases the activity of ALT, AST and ALP due to damage to liver cells and rupture of plasma membranes of these cells, the use of 200 mg of silymarin per kg of diet can reduce the activity of these enzymes in the blood of stressed ducks. This indicates the hepatoprotective properties of silymarin in protecting the plasma membrane of liver cells. It is thought that silymarin has anti‐inflammatory and antioxidant properties that can stimulate liver cell regeneration (Vargas Mendoza et al., 2014). Moreover, silymarin increases hepatic glutathione production by inducing cysteine synthesis and inhibiting its catabolism by taurine. The regulation of cysteine synthesis may subsequently help the antioxidant defence (Kwon et al., 2013). In addition, Miranda et al. (2000) reported that silymarin has antioxidant properties due to its polyphenolic structure, and its hydroxyl groups have the potential to neutralize free radicals. These mechanisms are consistent with our observations. So that the silymarin used in this experiment was able to increase the serum TAC. Higher TAC reduces the production of free radicals leading to a reduction in lipid peroxidation, which in turn reduces the level of MDA (an indicator of lipid peroxidation) concentration of serum and liver. These findings were supported by the results of liver histology and reduced relative expression of the TNF‐α gene in laying hens of the present study.

To further verify the effects of silymarin on hepatic redox status in laying hens, the mRNA abundance of associated genes in the liver was also evaluated in this study. Lipid oxidation in the liver is the main source of ROS production. Following the accumulation of triglycerides in the liver, oxidation of excess fatty acids leads to the overproduction of ROS and consequently oxidative stress in the liver (Zhu et al., 2018). Superoxide dismutase (SOD), GPx and catalase are the most important antioxidant enzymes that inhibit the formation of free radicals. These enzymes act as the primary defence system against ROS during oxidative stress (Zhu et al., 2018). In the present study, hepatic GPx activity was significantly higher compared to the control group. Thus, silymarin can reduce oxidative stress by increasing the activity of antioxidant enzymes. This may be related to the antioxidant properties of silymarin (Ma & Kinneer, 2002; Zhu et al., 2014). TNF‐α and IL‐6 are the best known mediators of the primary inflammatory response. ROS can increase the expression of genes such as iNOS, TNF‐α and IL‐1 (Ziamajidi et al., 2018). iNOS is significantly increased in response to inflammation and oxidative stresses. Under many pathological conditions, iNOS produces large amounts of NO, which can lead to liver cell damage (Madar et al., 2005). Therefore, each component that has the antioxidant capacity can reduce oxidative stress status and down‐regulate iNOS gene expression and NO level (Ziamajidi et al., 2018). In this study, the levels of TNF‐α, IL‐6 and iNOS in the liver were significantly lower compared to the control group, and these results showed that the use of silymarin is effective in reducing the inflammatory response.

It is reported that silymarin plays a vital role in the regulation of lipid metabolism and liver oxidative stress in a mouse model of the non‐alcoholic fatty liver. So that silymarin reduces the expression of FASN mRNA and ACACA (Ni & Wang, 2016). FASN is an enzyme that catalyses the synthesis of fatty acids and converts nutrients in the liver to fat to store energy in the body (Jensen‐Urstad & Semenkovich, 2012). ACACA is synthesized by malonyl‐CoA to bind acetyl coenzymes and produce fatty acids (Kang et al., 2020). Therefore, reducing the expression of FASN and ACACA genes in the present study indicates that silymarin prevents the accumulation of liver fat by regulating lipid metabolism. So, decreased liver fat and liver dry matter contents maybe through a decrease in the synthesis of fat. Moreover, a decreased liver weight could reflect a reduction of the percentage of liver fat, which has been shown in silymarin due to the ability of the drugs to counteract the cholesterol accumulation in the liver.

The hepatic protection of silymarin has been studied by researchers and attributed to its antioxidant mechanism for many years (Shaker et al., 2010). As regards non‐alcoholic fatty liver disease is mainly due to the accumulation of fat in the liver, we investigated the influence of silymarin on hepatic steatosis and lipid metabolism. Histological score and hepatic lipid level are the two most important factors in determining the severity of non‐alcoholic fatty liver disease (Ni & Wang, 2016). It has been reported that in the treatment of mouse model of the non‐alcoholic fatty liver, silymarin can induce hepatic steatosis and non‐alcoholic fatty liver disease activity score (steatosis grade + balloon grade + inflammation grade), because silymarin can regulate lipid metabolism and oxidative stress (Ni & Wang, 2016). In our study, silymarin was able to reduce hepatic steatosis, which was supported by the results obtained from the histopathological study and reducing the amount of fat in liver tissue. Silymarin acts as an antioxidant because it can not only prevent lipid peroxidation by scavenging free radicals but can also affect enzyme systems associated with glutathione and SOD (Podder et al., 2012).

In vitro, silymarin at high concentrations continuously enhances the stability of the hepatocellular plasma membrane (Basiglio et al., 2009). Lipid peroxidation is one of the main mechanisms that lead to the destruction of the cell membrane and the increase in liver disease (Hellerbrand et al., 2017). Under these conditions, the hepatoprotective effects of silymarin appear to depend on preventing lipid peroxidation (Kang et al., 2002), regulating membrane permeability and increasing membrane stability (Basiglio et al., 2009), and its anti‐inflammatory effects (Kim et al., 2012).

Moreover, the better results obtained with nano‐silymarin or lecithinized silymarin for the parameters measured in the present study can be explained by the findings that nanoparticles have different properties that facilitate their pharmacological behaviour in comparison to larger molecules (Yusuf et al., 2023). One of the most important applications of nanotechnology in medicine is in drug delivery systems. Most conventional drugs have poor bioavailability and poor aqueous solubility, which limits their absorption and retention in biological systems (Khadka et al., 2014), so nanoparticles are used to improve the efficacy of many traditional/conventional drugs. In addition, lecithin, which is a type of phospholipid, acts as vital components of the cell membrane to keep membrane fluidity and an absorption enhancer to facilitate drug absorption (Jin et al., 2013). Therefore, lecithin‐based formulations increase the bioavailability and improve the therapeutic efficacy of drugs with poor oral absorption (Yanyu et al., 2006). Lecithin can pass through cell membranes and facilitates encapsulated drug absorption due to its amphipathic nature (Chen et al., 2016). This means that it has both hydrophilic (water‐attracting) and hydrophobic (water‐repelling) regions, allowing it to interact with the lipid bilayer of the cell membrane and move across it. So, the nano‐silymarin and silymarin‐lecithin complexes used in this experiment were more bioavailable compared to the conventional drug owing to their faster release and an enhanced capacity to pass through the lipid‐rich biological membranes and finally reach the systemic circulation (Parveen et al., 2011).

## 5 CONCLUSIONS

Supplementing the diet of laying hens with silymarin significantly decreased the liver fat, dry matter and MDA contents by regulating the expression of FASN, GPx, IL‐6 and TNF‐α genes in the liver. As a high liver fat content is one of the predisposing factors for fatty liver syndrome, it was concluded that supplementing the diet with silymarin may be useful in the prevention of the disease, which in turn could improve the aged bird performance. In addition, in most cases, the effects were more significant when silymarin converted to NSM or administered as LSM due to their faster release and an enhanced capacity to pass through the biological membranes. Furthermore, from the results of this study, it can be concluded that silymarin especially with 200 mg/kg BW of NSM or LSM has a vital role in the regulation of lipid metabolism and oxidative stress.

## AUTHOR CONTRIBUTIONS

**Samira Faryadi**: Conceptualization; visualization; investigation; methodology; samples data collection; software and formal analysis; writing–original draft; review and editing; revision of the article and reply to the referee. **Ardashir Sheikhahmadi** and **Ayoub Farhadi**: Supervision; visualization; design; direction and funding acquisition; review and editing.

## CONFLICT OF INTEREST STATEMENT

The authors declare no conflicts of interest.

## FUNDING INFORMATION

None.

## ETHICS STATEMENT

All experimental procedures were conducted according to the international protocols and approved by Research Committee of the University of Kurdistan, Iran.

### PEER REVIEW

The peer review history for this article is available at https://publons.com/publon/10.1002/vms3.70025.

## Data Availability

Data available on request from the authors.
